# Interactive Tree Of Life (iTOL) v5: an online tool for phylogenetic tree display and annotation

**DOI:** 10.1093/nar/gkab301

**Published:** 2021-04-22

**Authors:** Ivica Letunic, Peer Bork

**Affiliations:** Biobyte Solutions GmbH, Bothestr 142, 69126 Heidelberg, Germany; EMBL, Meyerhofstrasse 1, 69117 Heidelberg, Germany; Department of Bioinformatics, Biocenter, University of Würzburg, 97074 Würzburg, Germany; Yonsei Frontier Lab (YFL), Yonsei University, Seoul 03722, South Korea

## Abstract

The Interactive Tree Of Life (https://itol.embl.de) is an online tool for the display, manipulation and annotation of phylogenetic and other trees. It is freely available and open to everyone. iTOL version 5 introduces a completely new tree display engine, together with numerous new features. For example, a new dataset type has been added (MEME motifs), while annotation options have been expanded for several existing ones. Node metadata display options have been extended and now also support non-numerical categorical values, as well as multiple values per node. Direct manual annotation is now available, providing a set of basic drawing and labeling tools, allowing users to draw shapes, labels and other features by hand directly onto the trees. Support for tree and dataset scales has been extended, providing fine control over line and label styles. Unrooted tree displays can now use the equal-daylight algorithm, proving a much greater display clarity. The user account system has been streamlined and expanded with new navigation options and currently handles >1 million trees from >70 000 individual users.

## Graphical Abstract

**Graphical Abstract:**
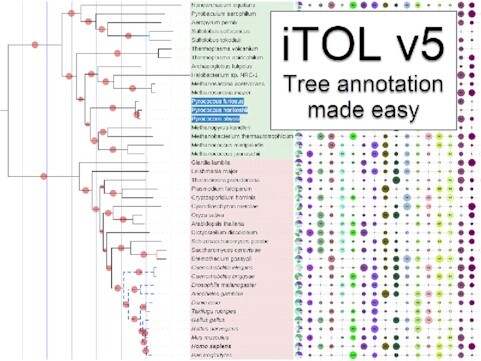
iTOL: an online tool for the tree display and annotation.

## INTRODUCTION

Phylogenetics and phylogenetic trees are heavily used in a wide variety of biological and other scientific studies, and classical tree visualization is supported by many software tools (1,2), including Interactive Tree Of Life (iTOL) (3), which introduced the annotation of trees with various types of additional data. Nowadays, various software packages offer additional tree annotation features, both online and as stand-alone packages or libraries, for example ETE toolkit (4), ggtree (5), Dendroscope (6), PhyD3 (7) or Evolview (8). Here, we report on the current state of iTOL as well as recent developments, which have expanded and streamlined its functionality.

## TREE DISPLAY

**Figure 1.**
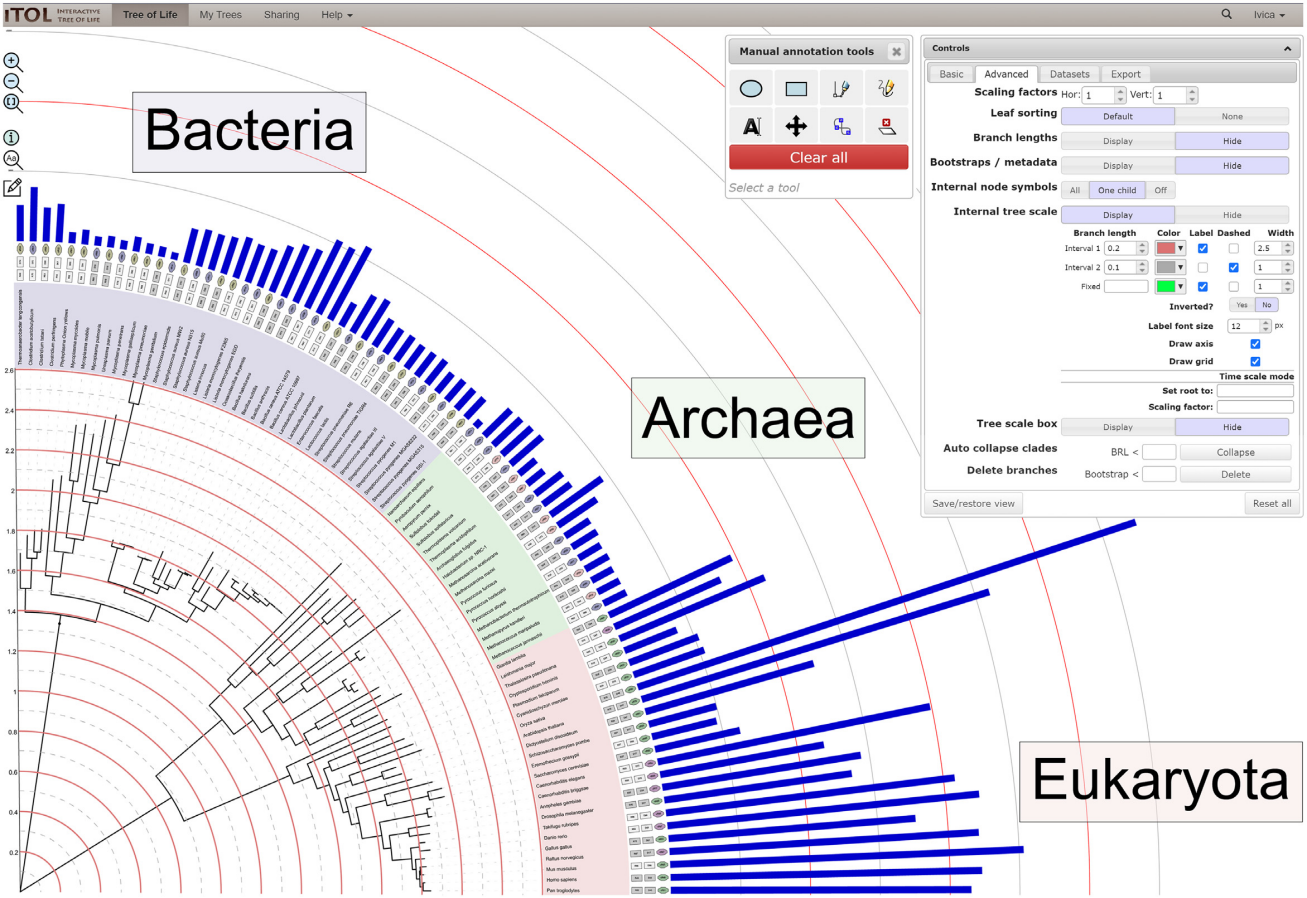
iTOL’s user interface. A phylogenetic tree annotated with several datasets is displayed, highlighting several new features. Internal tree scale level lines were individually styled, and an axis was added. Manual annotation drawing tools were used to add the taxonomic domain labels outside the tree. Manual annotations can be interactively scaled, rotated and repositioned.

iTOL is an online tool, accessible with any modern web browser (Figure 1). The tree display engine is implemented in pure JavaScript and uses the HTML5 Canvas element for visualization. Most of the display calculations and functionality are performed by the client web browser, allowing fine-grained interactive control over various display parameters. In iTOL v5, the display engine was completely rewritten and optimized with support for high-resolution displays. The low-level drawing functionality is now implemented through the paper.js library (https://paperjs.org).

### Input types and basic functions

iTOL supports commonly used phylogenetic tree formats such as Newick, Nexus (9) and phyloXML (10). Phylogenetic placement files created by EPA (11) and pplacer (12), as well as QIIME 2 trees and annotation files (13), are also supported.

All additional data used for various types of tree annotation are provided in plain text files, and simply dragged and dropped onto the trees visualized in the user’s web browser.

iTOL provides the most common functions available in any phylogenetic tree viewer. Various tree display formats are supported, such as phylograms or cladograms, rooted or unrooted, rectangular or radial. iTOL v5 introduces the ‘equal-daylight’ algorithm for the unrooted display mode, which tries to equalize the sizes of angular gaps between the tree’s clades. The resulting display shows a much better separation of nearby tree nodes and increases the overall clarity of the tree structure. On the other hand, it can cause internal subtree overlaps, as well as significant overlaps of leaf text labels. Users can select how many iterations of the equal-daylight algorithm to apply, or switch it off completely, and use the default ‘equal-angle’ unrooted tree display algorithm.

iTOL can manipulate the trees in various ways, and basic editing functions allow users to interactively delete or move single nodes or whole clades. Clades can also be pruned or collapsed, either manually or automatically, based on various parameters (such as associated bootstrap values or average branch length distances). iTOL v5 newly supports tree pruning through a simple text file with a list of tree nodes to include. Users can drop such files onto the display to automatically create a pruned tree representation.

Trees can be re-rooted manually on any node or automatically using the midpoint rooting method. Tree leaves can be sorted in various ways, either manually or automatically.

A complete overview of changes and functions added since the last publication (14) is listed on the iTOL’s version history page (https://itol.embl.de/version_history.cgi).

## TREE ANNOTATION

**Figure 2.**
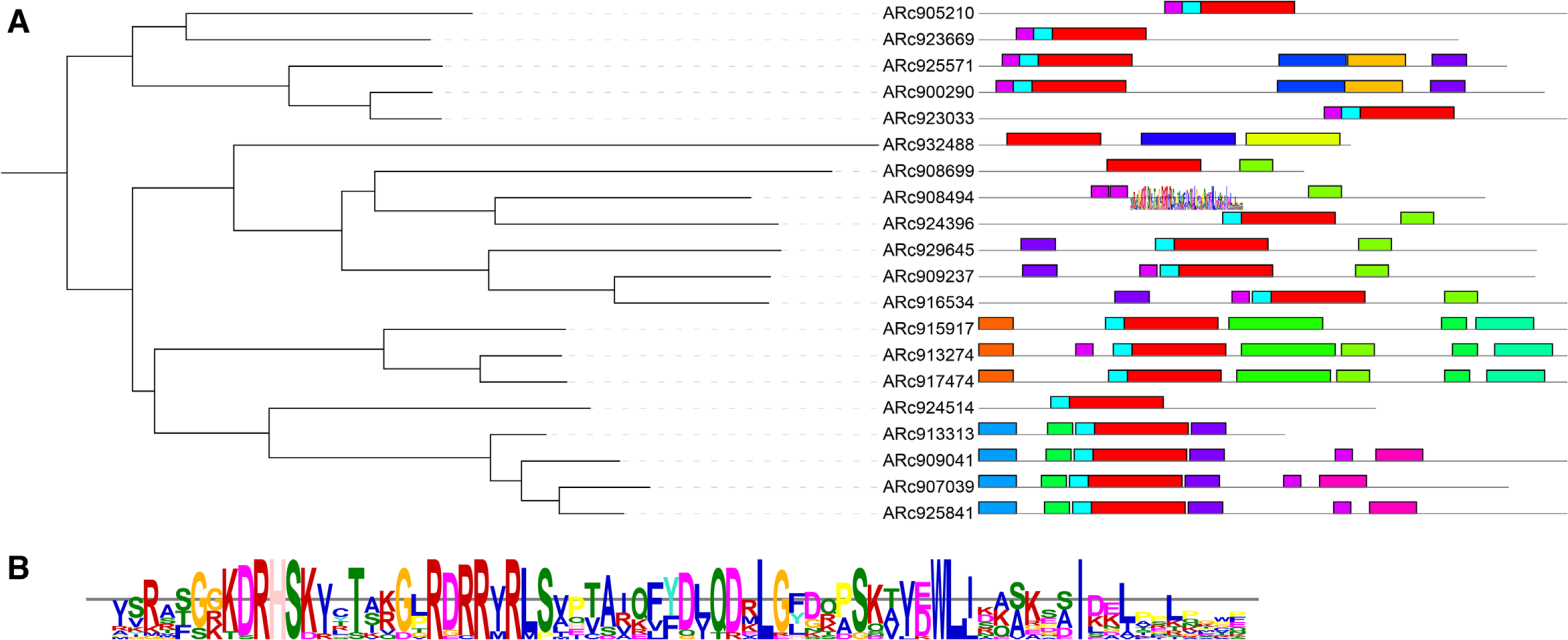
MEME motif dataset visualization in iTOL. (**A**) Dataset display: an XML file created by the MEME suite has been dropped onto the tree and visualized with a style similar to the default MEME results page. (**B**) MEME motif logo: zoomed in detail of the dataset, with two motifs displayed as MEME sequence logos, showing the individual residue probabilities as different sized letters.

iTOL v5 offers various new annotation features, extended functionality in the visualization of existing dataset types, and a new dataset type, MEME motifs (Figure 2). MEME datasets are used to visualize sequence motifs detected by the MEME suite (15). The datasets are created automatically by simply dropping the XML result files generated by the MEME suite onto the phylogenetic tree display in iTOL. The visualization style is similar to the default output generated by the MEME suite and uses the same colors and layout. Individual motifs can be displayed as MEME sequence logos, showing the individual residue probabilities as differently sized letters (Figure 2B).

### Displaying branch length, support and metadata values

iTOL v5 expands the node metadata visualization options with several new features. MRBAYES (16) and the New Hampshire X (NHX) formatted metadata support has been extended to support non-numerical values, which can be visualized as branch text labels or used to color the tree branches with user-defined category colors.

Bootstrap support and other numerical metadata values can now be displayed as percentages and scaled by a user-defined factor. In addition to the precise horizontal position along the branch, metadata text labels can now also be shifted vertically by a user-defined amount.

If multiple metadata values are available in the tree, these can all be displayed simultaneously as slash delimited text labels on the tree branches.

### Datasets and the interactive editor

iTOL currently supports the visualization of 19 different dataset types. Most of these datasets can be uploaded as plain text files, which use simple predefined templates that are available through iTOL’s help pages. The support for the external data types has been extended and now includes QIIME FeatureData[AlignedSequence] QZA files, which can be dropped onto the trees to automatically create multiple sequence alignment visualizations.

In addition to the template files, the most commonly used dataset types can be generated and edited directly in the interactive editor, within the iTOL web user interface. In iTOL v5, the editor has been streamlined and expanded with support for new dataset types. Raw data, as well as dataset legends, scales and other information, can be edited directly through a spreadsheet-like interface, with dynamically updating tree visualization.

Dataset support in the unrooted tree display mode has been extended, and now includes the text label datasets, as well as the newly introduced MEME motifs. Even though the individual dataset entries cannot be aligned in this mode, they still provide users with valuable annotation options.

### Tree and dataset scales and legends

Each dataset in iTOL can have an associated legend displayed next to the tree. In iTOL v5, all the legends are now draggable and can be manually positioned anywhere in the display, or their position can be predefined in the user-prepared dataset template files. Individual symbols in the legends can now be scaled. In addition, an automatically generated legend can be added to the tree when support values/metadata are visualized as symbols or branch colors.

Many dataset types in iTOL (like bar charts or protein domain architectures) can have a user-defined scale. The individual levels in any scale now support custom display labels with variable size, while the scale lines can be dashed and displayed in different widths.

iTOL’s internal tree scale display options have also been extended, with support for fixed position levels, in addition to the intervals. Individual scale level lines can be dashed and displayed in different widths. Internal tree scale can now be visualized as a simple axis and customized to display not only any values but also branch lengths. Users can specify a custom value for the tree root and a branch length scaling factor that are then used to calculate the scale text labels. This allows a simple display of timescales or similar features associated with the tree.

### Manual annotation tools

iTOL v5 introduces a set of basic manual drawing and labeling tools, allowing users to add simple shapes, polygons, lines and text labels anywhere on the tree display (Figure 1). These tools are not a replacement for a proper drawing or illustration software, but can help in adding simple additional annotations to the trees, simplifying the user's figure creation workflow and lowering the need for postprocessing.

## EXPORT

One of iTOL’s primary uses is the creation of high-quality figures for publication or inclusion into other documents. Due to the constantly increasing number of active users, the backend server has been extended to make the tree export faster. In peak usage times, the export can still be relatively slow, so we are actively developing a queueing system, which will prevent the overloading of the export server and make the process more stable for all users. We expect the queuing system to be fully implemented in the next major release of iTOL.

### Sharing exported tree figures

In addition to the standard tree export that downloads the created figure to the user computer, iTOL includes a system for the simple sharing of an unlimited number of figures. For each of their trees, users can create shared exported figures with individual descriptions and share a simple iTOL URL with their collaborators. The URL will display a table with an overview of all available shared exports for a tree, together with their format and description, allowing collaborators to simply access different tree figures created by the user. This feature can also be accessed through iTOL’s main data sharing page (https://itol.embl.de/sharing). Shared tree figures remain stored on the iTOL server and permanently accessible, unless removed by the user.

## SERVER ACCESS

iTOL is a self-sustaining tool and has been maintained and extended without any substantial public funding. In view of the constantly growing number of active users and uploaded trees, we have tried to find a sustainable model to maintain and further develop iTOL, serve the storage and CPU power needs, and provide timely technical support to our user base. Therefore, with version 5, albeit all tree annotation features remain freely available, certain account managing features of iTOL require an active subscription. Most user account management features also remain freely available apart from the saving of tree annotations to the iTOL server and the batch upload mode. User-uploaded trees and annotations remain permanently accessible, regardless of their subscription status.
